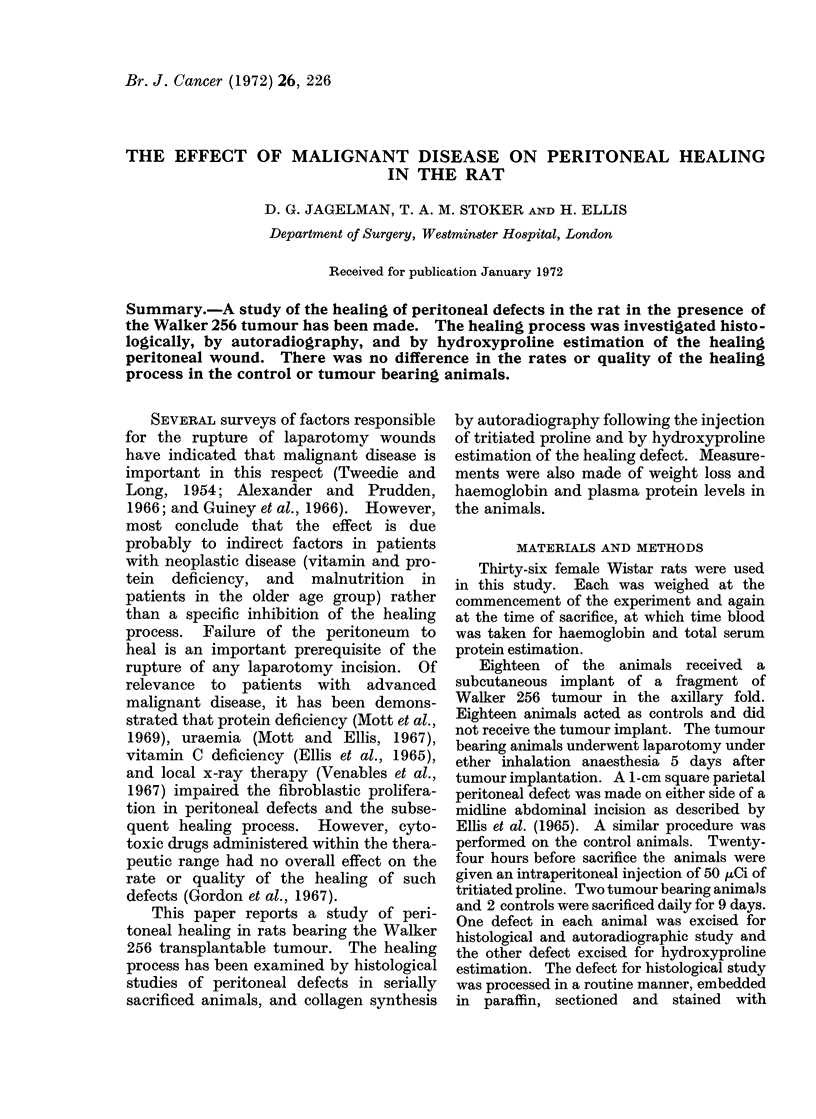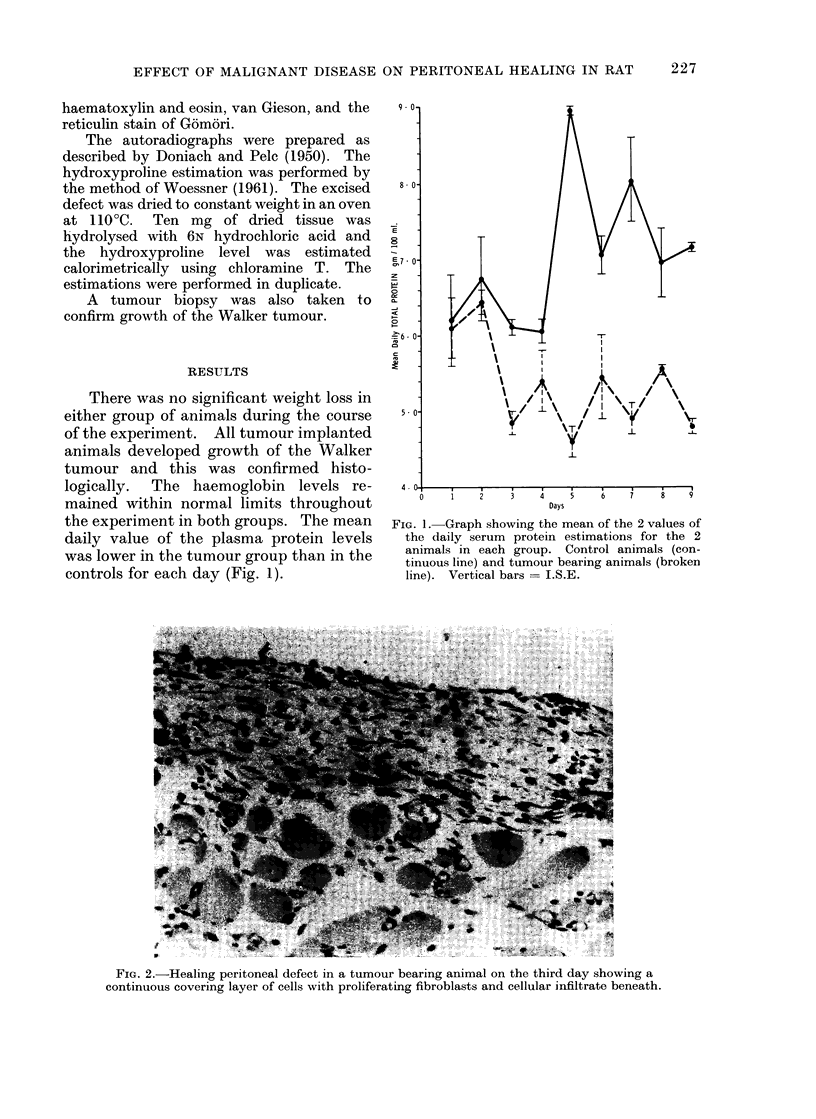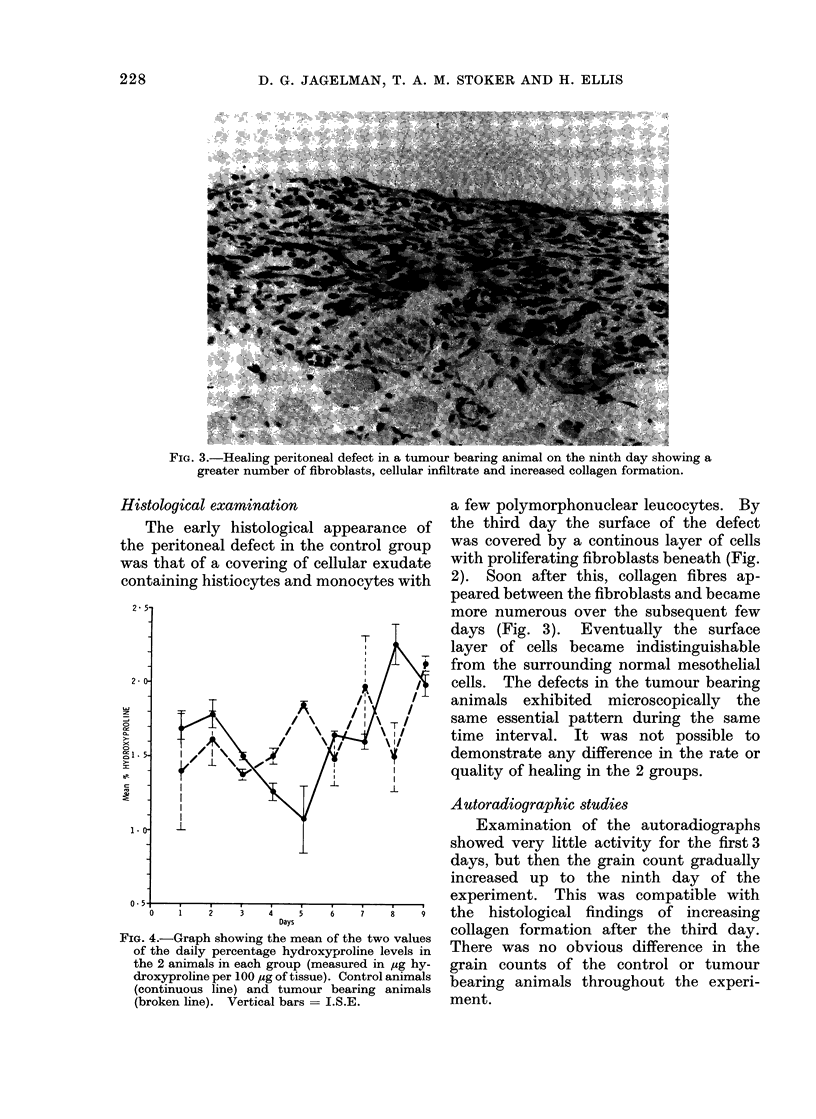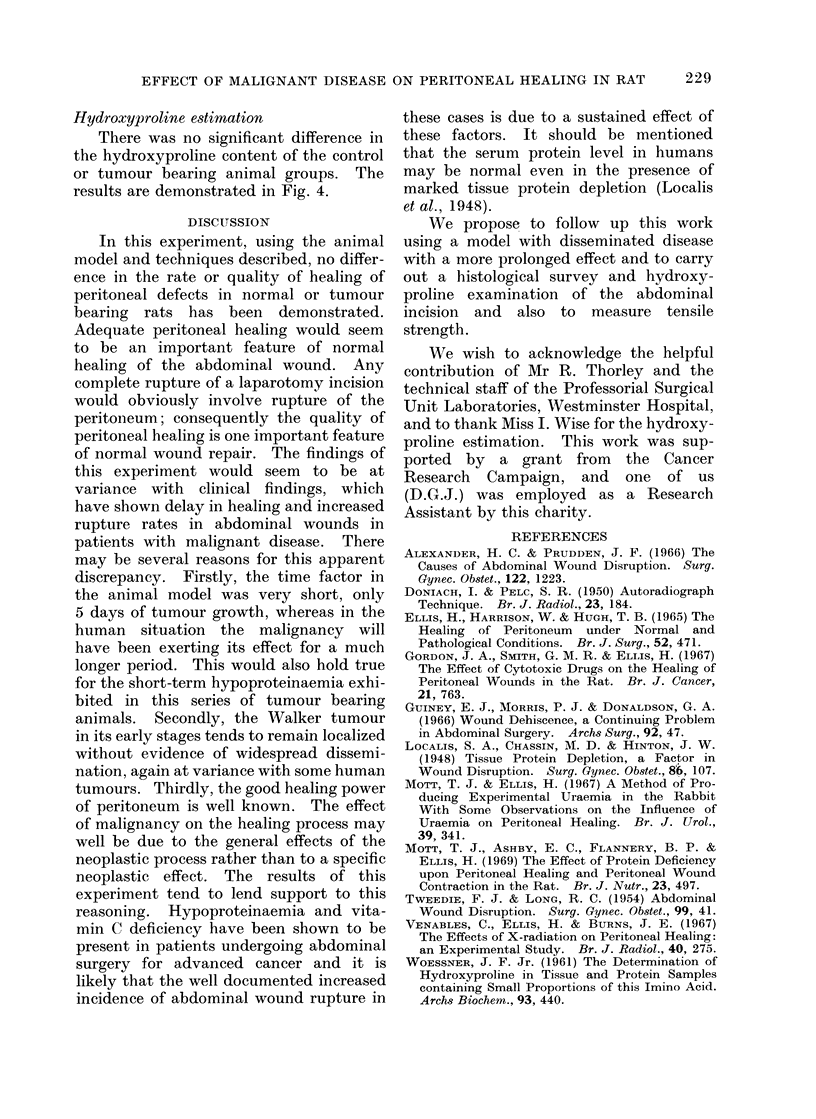# The Effect of Malignant Disease on Peritoneal Healing in the Rat

**DOI:** 10.1038/bjc.1972.30

**Published:** 1972-06

**Authors:** D. G. Jagelman, T. A. M. Stoker, H. Ellis

## Abstract

**Images:**


*Br. J. Cancer* (1972) **26**, 226

### THE EFFECT OF MALIGNANT DISEASE ON PERITONEAL HEALING IN THE RAT

D. G. JAGELMAN, T. A. M. STOKER AND H. ELLIS

*Department of Surgery, Westminster Hospital, London*

Received for publication January 1972

**Summary.—A study of the healing of peritoneal defects in the rat in the presence of the Walker 256 tumour has been made. The healing process was investigated histologically, by autoradiography, and by hydroxyproline estimation of the healing peritoneal wound. There was no difference in the rates or quality of the healing process in the control or tumour bearing animals.**

SEVERAL surveys of factors responsible for the rupture of laparotomy wounds have indicated that malignant disease is important in this respect (Tweedie and Long, 1954; Alexander and Prudden, 1966; and Guiney *et al.*, 1966). However, most conclude that the effect is due probably to indirect factors in patients with neoplastic disease (vitamin and protein deficiency, and malnutrition in patients in the older age group) rather than a specific inhibition of the healing process. Failure of the peritoneum to heal is an important prerequisite of the rupture of any laparotomy incision. Of relevance to patients with advanced malignant disease, it has been demonsstrated that protein deficiency (Mott *et al.*, 1969), uraemia (Mott and Ellis, 1967), vitamin C deficiency (Ellis *et al.*, 1965), and local x-ray therapy (Venables *et al.*, 1967) impaired the fibroblastic proliferation in peritoneal defects and the subsequent healing process. However, cytotoxic drugs administered within the therapeutic range had no overall effect on the rate or quality of the healing of such defects (Gordon *et al.*, 1967).

This paper reports a study of peritoneal healing in rats bearing the Walker 256 transplantable tumour. The healing process has been examined by histological studies of peritoneal defects in serially sacrificed animals, and collagen synthesis by autoradiography following the injection of tritiated proline and by hydroxyproline estimation of the healing defect. Measurements were also made of weight loss and haemoglobin and plasma protein levels in the animals.

### MATERIALS AND METHODS

Thirty-six female Wistar rats were used in this study. Each was weighed at the commencement of the experiment and again at the time of sacrifice, at which time blood was taken for haemoglobin and total serum protein estimation.

Eighteen of the animals received a subcutaneous implant of a fragment of Walker 256 tumour in the axillary fold. Eighteen animals acted as controls and did not receive the tumour implant. The tumour bearing animals underwent laparotomy under ether inhalation anaesthesia 5 days after tumour implantation. A 1-cm square parietal peritoneal defect was made on either side of a midline abdominal incision as described by Ellis *et al.* (1965). A similar procedure was performed on the control animals. Twenty-four hours before sacrifice the animals were given an intraperitoneal injection of 50 $\mu$Ci of tritiated proline. Two tumour bearing animals and 2 controls were sacrificed daily for 9 days. One defect in each animal was excised for histological and autoradiographic study and the other defect excised for hydroxyproline estimation. The defect for histological study was processed in a routine manner, embedded in paraffin, sectioned and stained with haematoxylin and eosin, van Gieson, and the reticulin stain of Gömöri.

The autoradiographs were prepared as described by Doniach and Pelc (1950). The hydroxyproline estimation was performed by the method of Woessner (1961). The excised defect was dried to constant weight in an oven at 110°C. Ten mg of dried tissue was hydrolysed with 6N hydrochloric acid and the hydroxyproline level was estimated calorimetrically using chloramine T. The estimations were performed in duplicate.

A tumour biopsy was also taken to confirm growth of the Walker tumour.

### RESULTS

There was no significant weight loss in either group of animals during the course of the experiment. All tumour implanted animals developed growth of the Walker tumour and this was confirmed histologically. The haemoglobin levels remained within normal limits throughout the experiment in both groups. The mean daily value of the plasma protein levels was lower in the tumour group than in the controls for each day (Fig. 1).

Fig. 1.—Graph showing the mean of the 2 values of the daily serum protein estimations for the 2 animals in each group. Control animals (continuous line) and tumour bearing animals (broken line). Vertical bars = I.S.E.

Fig. 2.—Healing peritoneal defect in a tumour bearing animal on the third day showing a continuous covering layer of cells with proliferating fibroblasts and cellular infiltrate beneath.

FIG. 3.—Healing peritoneal defect in a tumour bearing animal on the ninth day showing a greater number of fibroblasts, cellular infiltrate and increased collagen formation.

*Histological examination*

The early histological appearance of the peritoneal defect in the control group was that of a covering of cellular exudate containing histiocytes and monocytes with a few polymorphonuclear leucocytes. By the third day the surface of the defect was covered by a continous layer of cells with proliferating fibroblasts beneath (Fig. 2). Soon after this, collagen fibres appeared between the fibroblasts and became more numerous over the subsequent few days (Fig. 3). Eventually the surface layer of cells became indistinguishable from the surrounding normal mesothelial cells. The defects in the tumour bearing animals exhibited microscopically the same essential pattern during the same time interval. It was not possible to demonstrate any difference in the rate or quality of healing in the 2 groups.

FIG. 4.—Graph showing the mean of the two values of the daily percentage hydroxyproline levels in the 2 animals in each group (measured in $\mu$g hydroxyproline per 100 $\mu$g of tissue). Control animals (continuous line) and tumour bearing animals (broken line). Vertical bars = I.S.E.

*Autoradiographic studies*

Examination of the autoradiographs showed very little activity for the first 3 days, but then the grain count gradually increased up to the ninth day of the experiment. This was compatible with the histological findings of increasing collagen formation after the third day. There was no obvious difference in the grain counts of the control or tumour bearing animals throughout the experiment.

*Hydroxyproline estimation*

There was no significant difference in the hydroxyproline content of the control or tumour bearing animal groups. The results are demonstrated in Fig. 4.

### DISCUSSION

In this experiment, using the animal model and techniques described, no difference in the rate or quality of healing of peritoneal defects in normal or tumour bearing rats has been demonstrated. Adequate peritoneal healing would seem to be an important feature of normal healing of the abdominal wound. Any complete rupture of a laparotomy incision would obviously involve rupture of the peritoneum; consequently the quality of peritoneal healing is one important feature of normal wound repair. The findings of this experiment would seem to be at variance with clinical findings, which have shown delay in healing and increased rupture rates in abdominal wounds in patients with malignant disease. There may be several reasons for this apparent discrepancy. Firstly, the time factor in the animal model was very short, only 5 days of tumour growth, whereas in the human situation the malignancy will have been exerting its effect for a much longer period. This would also hold true for the short-term hypoproteinaemia exhibited in this series of tumour bearing animals. Secondly, the Walker tumour in its early stages tends to remain localized without evidence of widespread dissemination, again at variance with some human tumours. Thirdly, the good healing power of peritoneum is well known. The effect of malignancy on the healing process may well be due to the general effects of the neoplastic process rather than to a specific neoplastic effect. The results of this experiment tend to lend support to this reasoning. Hypoproteinaemia and vitamin C deficiency have been shown to be present in patients undergoing abdominal surgery for advanced cancer and it is likely that the well documented increased incidence of abdominal wound rupture in these cases is due to a sustained effect of these factors. It should be mentioned that the serum protein level in humans may be normal even in the presence of marked tissue protein depletion (Localis *et al.*, 1948).

We propose to follow up this work using a model with disseminated disease with a more prolonged effect and to carry out a histological survey and hydroxyproline examination of the abdominal incision and also to measure tensile strength.

We wish to acknowledge the helpful contribution of Mr R. Thorley and the technical staff of the Professorial Surgical Unit Laboratories, Westminster Hospital, and to thank Miss I. Wise for the hydroxyproline estimation. This work was supported by a grant from the Cancer Research Campaign, and one of us (D.G.J.) was employed as a Research Assistant by this charity.

### REFERENCES

Alexander, H. C. & Prudden, J. F. (1966) The Causes of Abdominal Wound Disruption. *Surg. Gynec. Obstet.*, **122**, 1223.

Doniach, I. & Pelc, S. R. (1950) Autoradiograph Technique. *Br. J. Radiol.*, **23**, 184.

Ellis, H., Harrison, W. & Hugh, T. B. (1965) The Healing of Peritoneum under Normal and Pathological Conditions. *Br. J. Surg.*, **52**, 471.

Gordon, J. A., Smith, G. M. R. & Ellis, H. (1967) The Effect of Cytotoxic Drugs on the Healing of Peritoneal Wounds in the Rat. *Br. J. Cancer*, **21**, 763.

Guiney, E. J., Morris, P. J. & Donaldson, G. A. (1966) Wound Dehiscence, a Continuing Problem in Abdominal Surgery. *Archs Surg.*, **92**, 47.

Localis, S. A., Chassin, M. D. & Hinton, J. W. (1948) Tissue Protein Depletion, a Factor in Wound Disruption. *Surg. Gynec. Obstet.*, **86**, 107.

Mott, T. J. & Ellis, H. (1967) A Method of Producing Experimental Uraemia in the Rabbit With Some Observations on the Influence of Uraemia on Peritoneal Healing. *Br. J. Urol.*, **39**, 341.

Mott, T. J., Ashby, E. C., Flannery, B. P. & Ellis, H. (1969) The Effect of Protein Deficiency upon Peritoneal Healing and Peritoneal Wound Contraction in the Rat. *Br. J. Nutr.*, **23**, 497.

Tweedie, F. J. & Long, R. C. (1954) Abdominal Wound Disruption. *Surg. Gynec. Obstet.*, **99**, 41.

Venables, C., Ellis, H. & Burns, J. E. (1967) The Effects of X-radiation on Peritoneal Healing: an Experimental Study. *Br. J. Radiol.*, **40**, 275.

Woessner, J. F. Jr. (1961) The Determination of Hydroxyproline in Tissue and Protein Samples containing Small Proportions of this Imino Acid. *Archs Biochem.*, **93**, 440.